# Chromosomal Evolution in the Phylogenetic Context: A Remarkable Karyotype Reorganization in Neotropical Parrot *Myiopsitta monachus* (Psittacidae)

**DOI:** 10.3389/fgene.2020.00721

**Published:** 2020-07-10

**Authors:** Ivanete de Oliveira Furo, Rafael Kretschmer, Patricia Caroline O’Brien, Jorge C. Pereira, Analía del Valle Garnero, Ricardo José Gunski, Rebecca E. O’Connor, Darren Karl Griffin, Anderson José Baia Gomes, Malcolm Andrew Ferguson-Smith, Edivaldo Herculano Correa de Oliveira

**Affiliations:** ^1^Instituto de Ciências Biológicas, Universidade Federal do Pará, Belém, Brazil; ^2^Laboratório de Cultura de Tecidos e Citogenética, Seção de Meio Ambiente, Instituto Evandro Chagas, Ananindeua, Brazil; ^3^Department of Veterinary Medicine, Cambridge Resource Centre for Comparative Genomics, University of Cambridge, Cambridge, United Kingdom; ^4^Departamento de Genética, Universidade Federal do Rio Grande do Sul, Porto Alegre, Brazil; ^5^School of Biosciences, University of Kent, Canterbury, United Kingdom; ^6^Animal and Veterinary Research Centre (CEVAV), University of Tràs-os-Montes and Alto Douro (UTAD), Vila Real, Portugal; ^7^Laboratório de Diversidade Genética Animal, Universidade Federal do Pampa, São Gabriel, Brazil; ^8^Instituto Federal do Pará, Abaetetuba, Brazil; ^9^Instituto de Ciências Exatas e Naturais, Universidade Federal do Pará, Belém, Brazil

**Keywords:** *Myiopsitta*, Arini tribe, phylogenetic, karyotype, rearrangements, breakpoints

## Abstract

*Myiopsitta monachus* is a small Neotropical parrot (Psittaciformes: Arini Tribe) from subtropical and temperate regions of South America. It has a diploid chromosome number 2*n* = 48, different from other members of the Arini Tribe that have usually 70 chromosomes. The species has the lowest 2*n* within the Arini Tribe. In this study, we combined comparative chromosome painting with probes generated from chromosomes of *Gallus gallus* and *Leucopternis albicollis*, and FISH with bacterial artificial chromosomes (BACs) selected from the genome library of *G. gallus* with the aim to shed light on the dynamics of genome reorganization in *M. monachus* in the phylogenetic context. The homology maps showed a great number of fissions in macrochromosomes, and many fusions between microchromosomes and fragments of macrochromosomes. Our phylogenetic analysis by Maximum Parsimony agree with molecular data, placing *M*. *monachus* in a basal position within the Arini Tribe, together with *Amazona aestiva* (short tailed species). In *M. monachus* many chromosome rearrangements were found to represent autopomorphic characters, indicating that after this species split as an independent branch, an intensive karyotype reorganization took place. In addition, our results show that *M*. *monachus* probes generated by flow cytometry provide novel cytogenetic tools for the detection of avian chromosome rearrangements, since this species presents breakpoints that have not been described in other species.

## Introduction

The order Psittaciformes comprises some 330–350 species, grouped into three families according to a new classification: Strigopidae, Cacatuidae and Psittacidae (Rheindt et al., 2014; Winkler et al., 2015). Within the Psittaciformes, the earliest divergence was between the New Zealand-restricted Strigopidae family (Kakapo, Kea, and Kaka) and other parrots, followed by the divergence between the Australasian cockatoos (Cacatuidae) and remaining parrots (Tavares et al., 2006; Wright et al., 2008). Recently, some studies have focused on the phylogenetic reconstruction of this order based on ancient geographic considerations. Wright et al. (2008) supported the hypothesis of an Australasian origin, during the cretaceous in Gondwana and afterward the division of the African and India/Madagascar block, with subsequent diversification through vicariance and dispersal.

Some studies have proposed the division of Psittacidae family into Old World and New World parrots (Miyaki et al., 1998). According to Tavares et al. (2006), the New world Parrots share a much earlier common ancestor with Australian parrots (59 million years ago, Mya), prior to the separation of Australia from Antarctica and South America.

Concerning chromosomal data, New World parrots (Arini Tribe) show a karyotype homogeneity when compared to Old World species, with a constant diploid chromosome number (2*n* = 70) (Caparroz and Duarte, 2004). However, some species have atypical diploid chromosome numbers, as low as 2*n* = 48 in *Myiopsitta monachus* (Furo et al., 2017) and 2*n* = 64 in *Graydidascalus brachyurus* (Caparroz and Duarte, 2004), or higher, as in *Forpus xanthopterygius* and *Brotogeris versicolurus* with 2*n* = 86 and 82, respectively (de Lucca et al., 1991; de Lucca and Marco, 2014).

Advances in molecular cytogenetics have shown that ancestral macrochromosomes have been highly rearranged in Psittaciformes (Nanda et al., 2007; Furo et al., 2015a, 2018). Hence, although chromosome painting with probes derived from chicken *Gallus gallus* (GGA) revealed three associations shared by all species of Psittaciformes analyzed so far – GGA1/4, GGA6/7, and GGA8/9 (Seabury et al., 2013; Furo et al., 2015a, 2018) – there are few chromosomal similarities shared between New World and Old World Psittaciformes.

Nevertheless, the results of GGA probes are limited as they do not detect most intrachromosomal rearrangements useful for phylogenetic inferences (Furo et al., 2015a, b; Furo et al., 2018; Kretschmer et al., 2018a). The use of probes from the white hawk (*Leucopternis albicollis*, LAL-2*n* = 66), with a highly derived karyotype involving multiple fissions on the ancestral chromosome (GGA1-GGA5) (de Oliveira et al., 2010), have enabled the detection of key intrachromosomal rearrangements (inversions, fissions), which together with no-reciprocal translocations and tandem fusions represent the main types of mechanisms responsible for their karyotypical divergence (Furo et al., 2015a, 2018).

*Myiopsitta monachus* (MMO) is a South American species (Higdon, 1998) with an atypical karyotype, with a diploid chromosome number 2*n* = 48, which is the lowest known diploid chromosome number among the Arini Tribe (Neotropical Psittacidae) and, together with the African species *Agapornis roseicollis*, has the lowest chromosome complement in the order (Nanda et al., 2007). Furthermore, *M*. *monachus* also atypical sex chromosomes, with an enlarged W chromosome, similar in size and morphology to the *Z*. These features are uncommon among Neornithes birds, which generally present heteromorphic sex chromosomes. The enlargement of the W is due to the accumulation and amplification of repetitive DNA (Furo et al., 2017).

A study by Tavares et al. (2006) based on mitochondrial and nuclear sequences, placed *M*. *monachus* in a clade together with *Amazona*, *Pionus*, *Pionopsitta*, *Triclaria*, and *Graydidascalus*, despite the fact that the *M*. *monachus* karyotype has been characterized only by Giemsa staining and microsatellite mapping (Furo et al., 2017).

Hence, in view of the value of whole chromosome painting in the improved understanding of chromosomal evolution, and the fact that *M*. *monachus* has an atypical chromosome complement in which the homologies with other species is still unknown, the aim of this study has been (a) to investigate the mechanisms underlining the origin of the low 2*n* of *M. monachus* by using GGA BAC probes and whole chromosome painting probes from *G. gallus* and *L. albicollis*; (b) to compare the karyotypes of *M. monachus* and members of Psittaciformes, to better determine their phylogenetic relationships. Additionally, we show that the use of probes derived from species with highly derived karyotypes such as *M. monachus* provides greater resolution in detecting interchromosomal rearrangements.

## Materials and Methods

### Samples, Cell Culture, and Chromosome Preparation

The experiments followed ethical protocols and were approved by the ethics committee (CEUA-Universidade Federal do Pará) under no. 170/2013 and SISBIO 68443-1. Metaphases were obtained from fibroblast culture of skin biopsies and feather pulp of 1 (female) individual of *Ara macao* (AMA) and 5 (3 males and 2 females) of *M*. *monachus* (Table 1), following Sasaki et al. (1968) with modifications. The samples were firstly fractionated mechanically in a Petri dish and afterward were incubated in a solution of Collagenase type IV for dissociation. Cells were then cultivated in DMEM (GIBCO) supplemented with calf bovine serum 20%, Aminiomax^TM^-II 5% and Penicillin (PNS) 1%, and incubated at 37°C. Chromosome suspensions were obtained using colcemid (Gibco, 100 μl for 5 ml of complete medium) followed by a treatment in hypotonic solution (KCl 0,075 M) and fixation in Carnoy’s fixative methanol: acetic acid (3:1 v/v).

**TABLE 1:** List of specimens collected in the present study.

Species	Number of individuals/sex	City/state
*Ara macao*	1 female	Belém/Pará-PA
*Myiopsitta monachus*	3 males and 2 females	Sapucaia do Sul/Rio Grande do Sul-RS

### Flow Sorting and Generation of Chromosome-Specific Painting Probes

Chromosome preparations for flow cytometry were obtained from a fibroblast cell line of a male *M*. *monachus*. The flow sorting followed the method described by Nie et al. (2015). Chromosomes were stained with chromomycin A3 (40 μg/ml, Sigma) and Hoechst 33258 (2 μg/ml, Sigma). Sorting was performed using a dual-laser cell sorter (MoFlo, Beckman Coulter). The primary sorted chromosome material, i.e., macro- and microchromosomes of *M. monachus* (hereafter designated as MMO) was amplified by degenerate oligonucleotide-primed polymerase chain reaction (DOP-PCR) (Telenius et al., 1992) and the resulting products were then labeled with biotin-16-dUTP or digoxigenin-dUTPs during secondary DOP-PCR amplification. The identity of probes was validated by their hybridization back to the metaphases of the original species. Because many chromosomes of MMO have similar sizes (making their identification difficult) we used reciprocal chromosome painting between MMO and *Ara macao* (AMA) to resolve any ambiguity in the chromosomal assignment of each flow peak and, in particular, of the peaks that contained two chromosomes. As molecular karyotype characterization of *Ara macao* is known, the use of this species helped to clarify the assignment of some MMO peaks.

### Preparation of Chicken BAC Clones and FISH

The bacterial artificial chromosomes (BACs) containing fragments from chromosomes 17–28, were labeled by Nick translation, fluorescein isothiocyanate-12-UTP (FITC-dUTP) (p-arms) (Roche) and Texas Red-12-dUTP (q-arms) (Invitrogen) and used in fluorescent *in situ* hybridization experiments following O’Connor et al. (2018). The slides were analyzed with an Olympus BX-61 epifluorescence microscope equipped with a cooled CCD camera and appropriate filters. Images were captured using SmartCapture3 (Digital scientific United Kingdom).

### Chromosome Painting With *G*. *gallus* and *L*. *albicollis* Probes

Sets of chromosome specific probes from *G*. *gallus* and *L*. *albicollis* were previously generated by flow-sorting of chromosomes in Cambridge Resource Center for Comparative Genomics (Cambridge, United Kingdom). We used a set of chicken chromosome probes with pairs 1–14. Comparative chromosome painting with *L*. *albicollis* probes used pairs homologous to chromosomes: GGA1 (LAL3, 6, 7, 15, and 18), GGA2 (LAL2, 4, and 20), GGA3 (LAL9, 13, 17, and 26), GGA4 (LAL1 and 16), GGA5 (LAL5) and GGA6 (LAL3) (de Oliveira et al., 2010). The protocols for hybridization followed de Oliveira et al. (2010). The slides were analyzed using a Zeiss Axioplan2 fluorescence microscope and ISIS software (Metasystems). Comparisons were based on the avian putative ancestral karyotype (PAK), in which pairs PAK 1–11 and PAK13-15 corresponded to GGA1-GGA3, GGA4q, GGA5-GGA9, GGA4p, GGA10, GGA12, GGA13, and GGA14, respectively (Griffin et al., 2007; Kretschmer et al., 2018a).

### Phylogenetic Analysis Using Chromosomal Characters

A binary matrix was constructed showing the presence and absence of discrete characters through chromosomal rearrangements and chromosome homologies among MMO and other species previously reported: *Amazona aestiva*, *Pyrrhura frontalis* (PFR), *Anodorhynchus hyacinthinus* (AHY), *Ara chloropterus* (ACH) and *Ara macao* (AMA) from the Neotropical Psittacidae (Seabury et al., 2013; Furo et al., 2015a, 2018). As outgroup, we used *G*. *gallus* and *Turdus rufiventris* (TRU) (Supplementary Table S1) (Kretschmer et al., 2014). The matrix generated was used in a parsimony cladistic analysis, using PAUP 4.0b10 (Phylogenetic Analysis Using Parsimony). A heuristic search to find the most parsimonious tree(s) was performed using Tree Bisection Reconnection (TBR) branch-swapping; the posterior bootstrap probability was obtained with one thousand replicates. For homology analysis, we used the nomenclature of the Putative Avian Ancestral Karyotype (PAK) (Griffin et al., 2007). Conclusions concerning chromosomal rearrangements and divergence time of species were traced by comparing our phylogenetic data with previously published data from Tavares et al. (2006).

## Results

### Karyotype and Chromosome Mapping With *G. gallus* and *L. albicollis* Whole-Chromosome Probes

**FIGURE 1:**
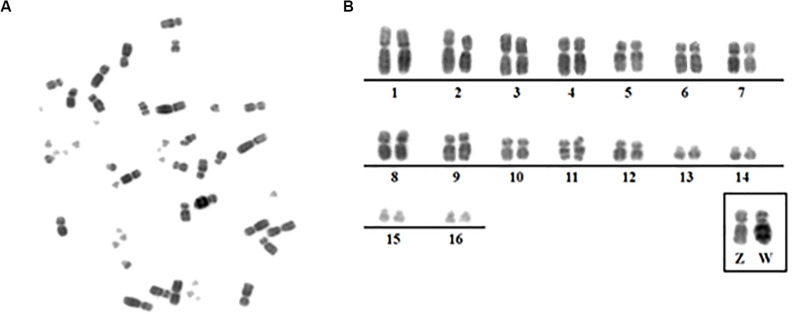
**(A)** Metaphase from the *Myiospitta monachus* female. **(B)** Karyotype from *Myiopsitta monachus* (2*n* = 48) showing only macrochromosomes 1–16 and ZW.

The chromosome number and morphology for the species analyzed here confirmed previous results: 2*n* = 68 in *A*. *macao* (AMA) and 2*n* = 48 in *M*. *monachus* (MMO) (Seabury et al., 2013; Furo et al., 2017; Figure 1).

*Gallus gallus* whole chromosome probes corresponded to 21 homologous segments in the MMO genome, and revealed fissions in several ancestral pairs, and fusions involving the segments that were fissioned. The correspondences found were: GGA1 (MMO3q, 4p/q, 12, 14q), GGA2 (MMO 2, 3p, 13q), GGA3 (MMO5 and MMO7), GGA4 (MMO1q/p), GGA6 (MMO 9q, 10q), GGA7 (MMO 9q, 10q), GGA8 (MMO 8p), GGA9 (MMO 8q), GGA10 (MMO 9p, 11), GGA13 (MMO 13p), GGA14 (MMO1p).

**FIGURE 2:**
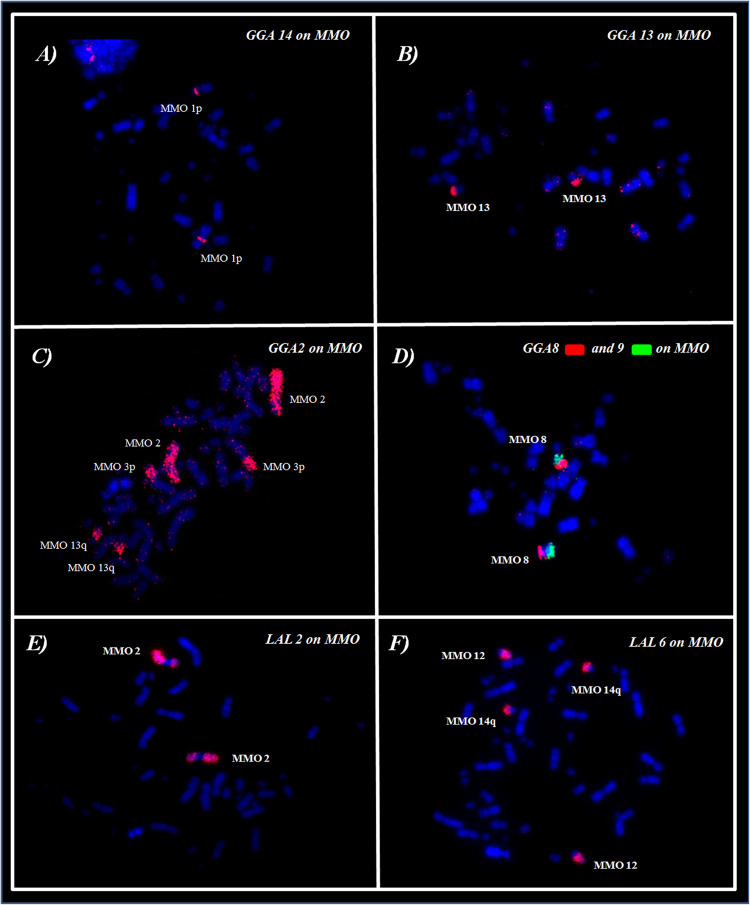
FISH using whole-chromosome probes of **(A–D)** *G*. *gallus* and **(E,F)** *L*. *albicollis* on *M*. *monachus*.

The fusions detected were: GGA1/GGA2, in MMO3; GGA3/GGA4 in MMO7p, GGA8/GGA9 in MMO8 and GGA2/GGA13 in MMO13. In addition, we observed the fusion between GGA6/GGA7, as observed in all members of Arini Tribe (Seabury et al., 2013; Furo et al., 2015a, 2018). However, due to an additional rearrangement in *M*. *monachus*, this association originated two distinct pairs: MMO9 (micro/GGA7/GGA6) and MMO10 (micro/GGA6/GGA7) (Figures 2A–D and Table 2).

**TABLE 2:** Chromosomal correspondence, avian putative ancestral karyotype (PAK), *G. gallus* (GGA) and *M. monachus* (MMO) chromosomes.

Ancestral chr.	Probes	Number of pairs	Pairs in MMO
PAK1	GGA1	4 Pairs	3q,4p/q,12,14q
PAK2	GGA2	3 pairs	2,3p,13q
PAK3	GGA3	2 pairs	5,7
PAK4	GGA4q	2 pairs	1q/7q
PAK5	GGA5	1 pair	6
PAK6	GGA6	2 pairs	9q,10q
PAK7	GGA7	2 pairs	9q,10q
PAK8	GGA8	1 pair	8p
PAK9	GGA9	1 pair	8q
PAK10	GGA4p	2 pair	1p, 7q
PAK11-13	GGA10-12	2 pairs	9p,11
PAK12	GGA11		
PAK14	GGA13	1 pair	13p
PAK15	GGA14	1 pair	1p

**FIGURE 3:**
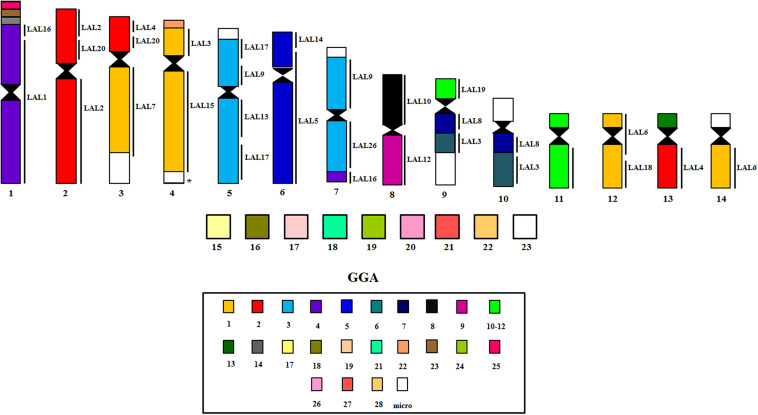
Homology maps with GGA, LAL probes and chicken BACs from microchromosomes on chromosomes of *M*. *monachus*.

The experiments using LAL probes allowed the detection of inversions involving the ancestral chromosomes 1–5 (Figures 2E,F). In *M. monachus* an unusual rearrangement was detected with LAL probes, in this case, LAL16 (homologous to GGA4p), which has fissioned resulting in two segments, one of which fused with GGA4q and the other GGA3. The homology maps of *M*. *monachus*, *G*. *gallus* and *L*. *albicollis* by chromosome painting are shown in Figure 3 and Table 2.

### Inferring Homeologies by BAC-FISH With GGA Probes Mapped Onto *M. monachus* Chromosomes

**FIGURE 4:**
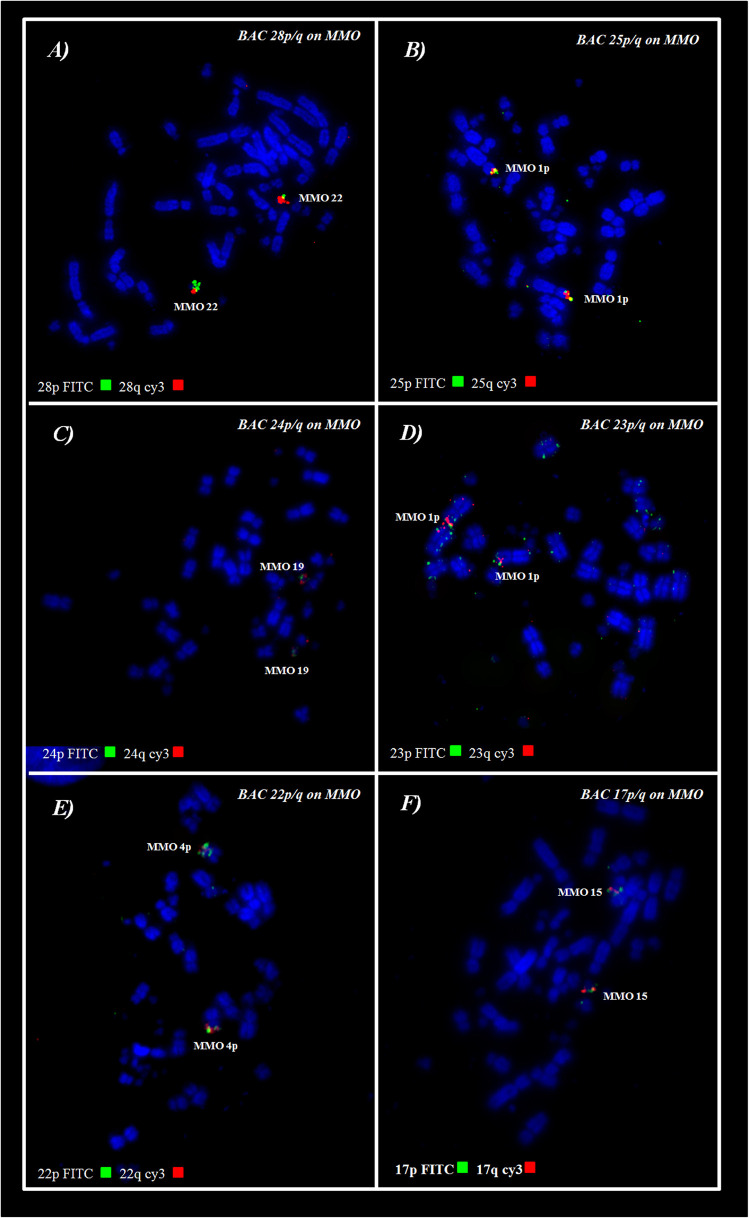
FISH using Chicken BACs from microchromosomes on *M*. *monachus*. **(A,C)** Examples of conserved microchromosomes. **(B,D–F)** Examples of microchromosomes involved in fusions with macrochromosomes. Red signals represent probes labelled with Cy3; Green signals represent probes labelled with FTIC.

BAC probes corresponding to GGA chromosome pairs 17–28, except pair 20 (which did not produce reproducible results) revealed the occurrence of many tandem fusions between microchromosomes and also fusions between macrochromosomes and microchromosomes as revealed in MMO1 (GGA4p/q+GGA14+GGA23+GGA25) and MMO4 (GGA1+GGA22) (Figures 3, 4 and Table 3).

**TABLE 3:** Chromosomal correspondence between chicken BACs from microchromosome (GGA) in *M. monachus* (MMO).

GGA Chr.	BAC Name	MMO Chr.
17q	CH261-42P16	15q
17p	CH261-113A7	15q
18p	CH261-60N6	16p
18q	CH261-72B18	16q
19p	CH261-10F1	17p
19q	CH261-50H12	17p
21q	CH261-122K8	18q
21p	CH261-83I20	18p
22q	CH261-18G17	4p
22p	CH261-40J9	4q
23p	CH261-191G17	1p
23q	CH261-90K11	1p
24p	CH261-103F4	19p
24q	CH261-65O4	19q
25q	CH261-127K7	1p
25p	CH261-59C21	1p
26q	CH261-170L23	20q
26p	CH261-186M13	20p
27q	CH261-28L10	21p
27p	CH261-66M16	21q
28p	CH261-64A15	22p
28q	CH261-72A10	22q

### Flow Karyotype of *M*. *monachus*

**FIGURE 5:**
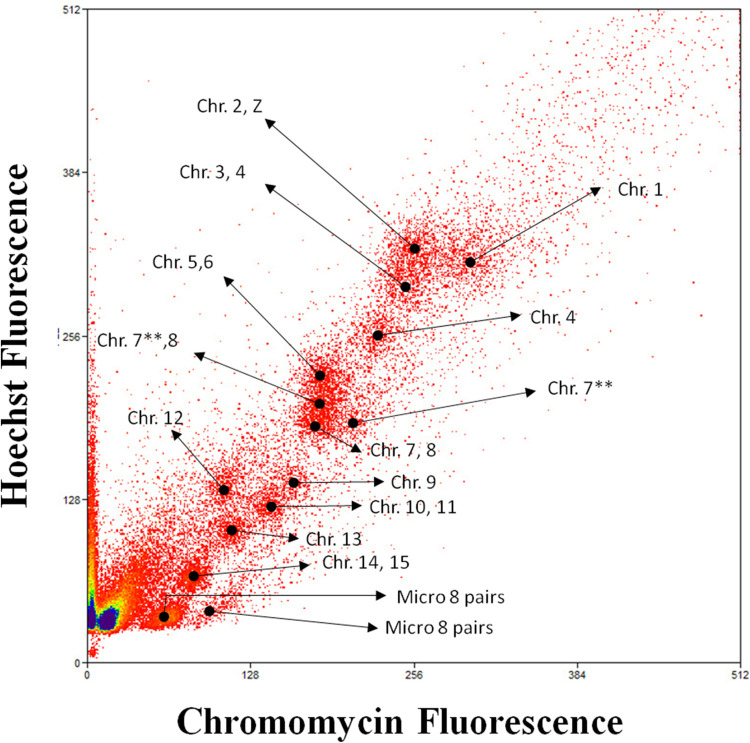
The bivariate flow karyotype of the *Myiopsitta monachus*. Chromosomes were sorted for DNA content and AT to GC base pair rations into 15 peaks after staining with Hoechst 22358 (vertical axis) and chromomycin-A (horizontal axis). Legend: **Different peaks contain same chromosomes.

**FIGURE 6:**
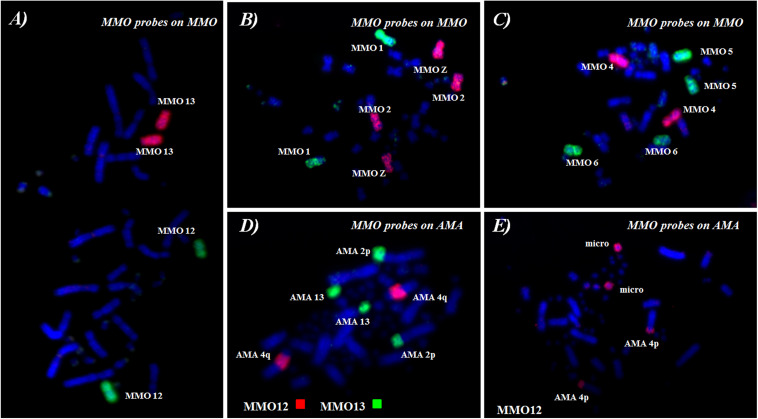
FISH using whole-chromosome probes [MMO **(A–C)**] onto *M*. *monachus* and [MMO **(D,E)**] *M*. *monachus* on *A*. *macao.*

The 48 chromosomes of *M*. *monachus* were resolved into 15 peaks by flow cytometry (Figure 5). The chromosomes in each peak of the flow karyotype were identified on MMO metaphases using FISH with labeled peak-specific DNA (Figures 6A–C). MMO chromosomes 1, 4, 9, 12, and 13 pairs formed a separate peak each. However, chromosomes 2+Z were found in the same peak as well as chromosomes 3+4, 5+6, 7+8, 10+11, and 14+15 and microchromosomes 15–23. However, some chromosomes were found in more than one peak, such as MMO 4, 7, and 8, and a possible explanation is the presence of repetitive DNA in some chromosomes of MMO, with a significant difference in the number and overall GC content of repeats between the homologs of some pairs, as described by Furo et al. (2017). These heteromorphisms would be sufficient to separate the homologs into different positions in the flow-karyotype (Figure 5).

### Chromosome Painting Between *M*. *monachus* and *A*. *macao*

**FIGURE 7:**
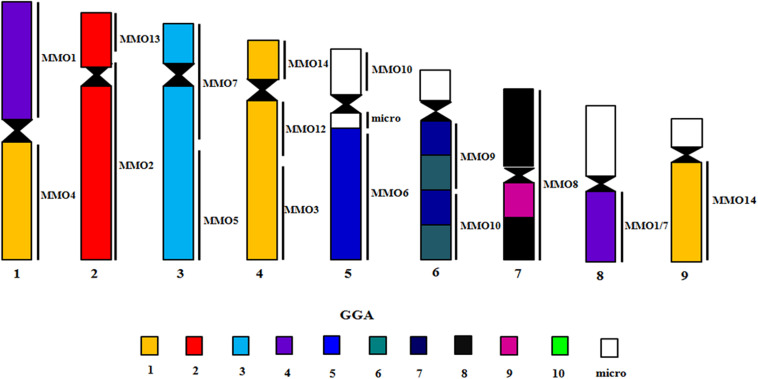
Homology map with GGA and MMO probes on metaphase chromosomes of *A*. *macao.* The results with GGA probes were obtained from Seabury et al. (2013).

Reciprocal chromosome painting between MMO and AMA established chromosome homologies between these species and defined the chromosome content of MMO peaks. FISH examples are shown in Figures 6D,E, and results of reciprocal chromosome painting between MMO and AMA are summarized in the karyotype of AMA (Figure 7 and Table 4).

**TABLE 4:** Chromosomal correspondence between *M. monachus* (MMO), *G. gallus* (GGA) and *A. macao* (AMA) chromosomes.

Chr MMO	GGA	Chr AMA
2, Z	2q, Z	2q, Z
1	4q, 4p,14,23,25,28	1p,8q + 4 micros
2, 3, 4, Z	2p/q,1p/q, Z, micros	1q,2q/p,4q,Z, micros
4	1p	1q
5,6	3p/q,5	3q,5q
5,6,7,8	3p/q,5,8,9	3,5,6,
8,9	7,6,8,9	6,7
7	3q	6
10,11	6,7,10,micro	7,micro
12	1q	4q
13	2p + micro	2p + micro
14,15	1q (MMO14), micro	1q (MMO14), micro

### Phylogenetic Analysis

Two most parsimonious trees were obtained using PAUP (Figure 10). The consensus tree presented score 37, and was obtained from 34 characters, 12 of them being phylogenetically informative. The tree length was 37, consistency index and homoplasic index was 0,9189 and 0,0811, respectively. The Psittaciformes are well supported as a monphyletic group, with high bootstrap support (100). In the basal branch is *A. aestiva*, followed by a branch including the other membership of Arini tribe (bootstrap of 69), the first species to split within this clade was MMO, followed by a branch (bootstrap 74) that contain the sister groups AHY/PFR and AMA/ACH, with bootstrap support of 63 and 50, respectively. The low bootstrap support in some branches were due to the low number of informative characters (numbers below of the branches), as most chromosome characters were autopomorphies, mainly due to intense chromosomal reshuffling observed in some species, such as *M. monachus*. In addition, some chromosome rearrangements could not be included because there is no information concerning chromosome homology involving the microchromosomes, with the exception of MMO.

## Discussion

### Chromosome Evolution in Psittaciformes

The advances in the last decades in comparative cytogenetics have clarified many aspects of the dynamic organization of avian genomes, mainly thanks to the use of *G. gallus* probes in many species belonging to different orders. For instance, it was possible to identify that their ancestral karyotype structure was kept stable over millions of years of evolution in many branches (Kretschmer et al., 2018a). This conservatism seems to count not only for macrochromosomes but also microchromosomes, as revealed by recent studies with chicken BACs from microchromosomes (O’Connor et al., 2018). Despite this, some avian groups such as Charadriiformes, Accipitriformes and Psittaciformes, exhibit highly reorganized karyotypes (de Oliveira et al., 2005, 2010; Nie et al., 2009; Furo et al., 2015a, 2018).

Important insights into chromosomal diversification in Psittaciformes have been highlighted by earlier studies with sets of *G. gallus* and *L. albicollis* whole-chromosome probes. According to a recent study by Furo et al. (2018), the chromosomal synapomorphies found in Psittaciformes, such as the associations between different pairs of the putative ancestral karyotype (PAK), such as PAK1/PAK4 (GGA1/GGA4q), PAK6/PAK7 (GGA6/GGA7) and PAK8/PAK9 (GGA8/GGA9) demonstrate a closer phylogenetic relationship among some genera of Neotropical Psittacidae. In addition, the data from the present study do not support tail size as a relevant taxonomic criterion for classifying this group, as proposed by some authors (Montón, 1977; Miyaki et al., 1997, 1998; Sick, 1997; Francisco and Galetti, 2001).

So far, all species of the Neotropical Psittacidae analyzed by chromosome painting showed homogeneity in the diploid chromosome number, corresponding generally to 2*n* = 70, however, *M. monachus* 2*n* = 48, represents an exception within this group. In fact, *M*. *monachus* shares the lowest 2*n* among Psittaciformes together with the African species *A*. *roseicollis* (ARO) (African Psittacidae) (Nanda et al., 2007). Nevertheless, phylogenetic studies, including our chromosome painting data, reveal that this similarity was limited to the chromosome number only. This happened because of the high rate of karyotype repatterning that occurred independently during their evolution, as observed in this study using *G*. *gallus* probes. Hence, the chromosome count is not always indicative of phylogenic proximity as in, for instance, *Agapornis* which is related to the Loriini (represented by *Lorius*) from Indonesia (Rheindt et al., 2014).

Likewise, the utilization of *L*. *albicollis* probes in *M*. *monachus* allowed us to identify a great number of pericentric and paracentric inversions. This is in agreement with the view that chromosomal rearrangements (inversions, fissions), which together with no-reciprocal translocations and tandem fusions represent the main rearrangements associated with karyotype evolution in Psittaciformes (Seabury et al., 2013). The methods used, however, are unable to distinguish inversions from centromeric repositioning (Rocchi et al., 2012).

**FIGURE 8:**
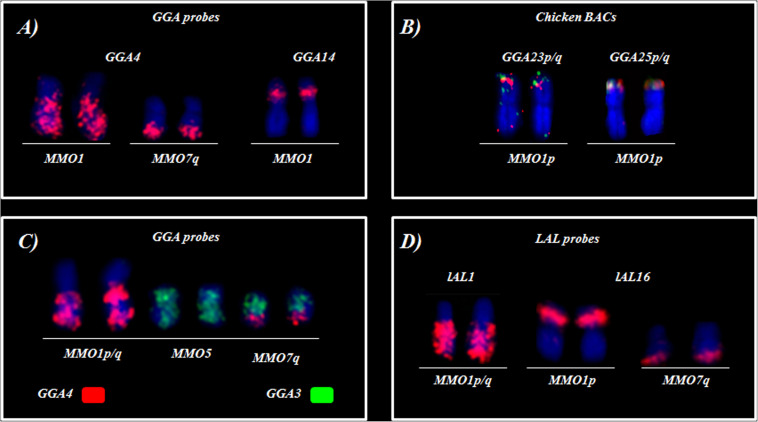
Summary of chromosome rearrangements involving GGA4 chromosome in *M*. *monachus*. **(A,C)** *G*. *gallus* probes **(B)** Chicken BACs **(D)** *L*. *albicollis* probes.

**FIGURE 9:**
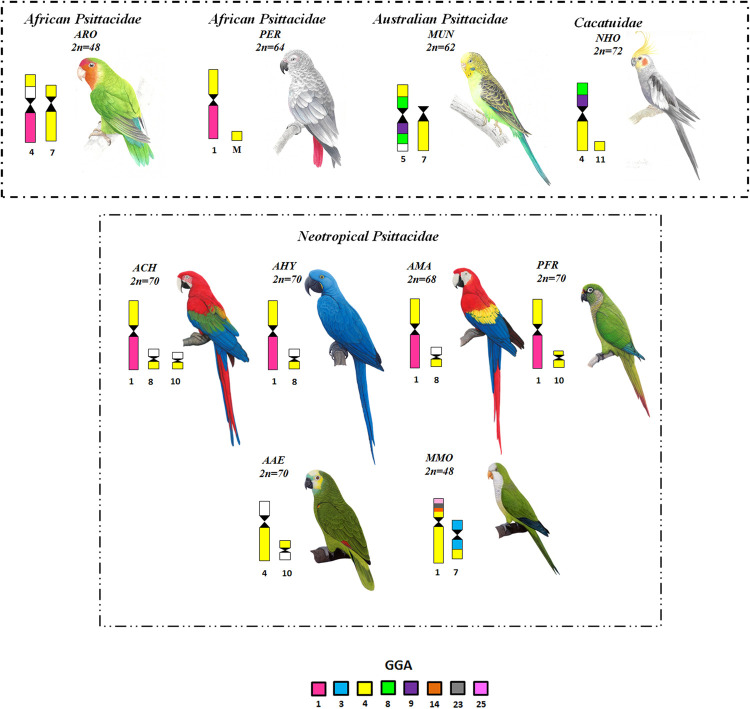
Representation of chromosome organization of four ancestral chromosomes in Psittaciformes, obtained by chromosome painting with *G. gallus* probes and chicken BACs from microchromosomes. Legend: GGA (*Gallus gallus*), ARO (*Agapornis roseicollis*), PER (*Psittacus erithacus*), MUN (*Melopsittacus undulatus*), NHO (*Nymphicus hollandicus*), ACH (*Ara chloropterus*), AHY (*Anodorhynchus hyacinthinus*), AMA (*Ara macao*), PRF (*Pyrrhura frontalis*), AAE (*Amazona aestiva*), MMO (*Myiopsitta monachus*).

In general, the ancestral macrochromosomes are involved in many fissions and fusions in *M*. *monachus*, several representing autapomorphies. For instance, an unusual rearrangement in MMO is the fusion between a small segment of PAK10 (GGA4p) with PAK4 (GGA4q). Generally, GGA4 (PAK4 and PAK10) corresponds to two pairs in most species of birds, belonging to different orders, such as Galliformes, Anseriformes, Passeriformes, Falconiformes, Strigiformes and Struthioniformes (Guttenbach et al., 2003; Kretschmer et al., 2014, 2015, 2018b). However, in *M*. *monachus* and other species of this order, these chromosomes are fused with other elements as shown in Figure 9 (Nanda et al., 2007; Seabury et al., 2013; Furo et al., 2015a, 2018). In *M*. *monachus* a fusion between these segments followed by fission in a region of PAK10 (GGA4p) was observed, nevertheless, the breakpoints of the segments corresponding to PAK10 (GGA4p) were not found in other species so far, because majority of molecular cytogenetic studies in birds has been limited to the use of *G*. *gallus* probes. It is thus not possible to know whether this peculiarity is exclusive to this species (Figure 8).

In addition, although we found the fusion of PAK6 (GGA6)/PAK7 (GGA7) in the karyotype of *M*. *monachus*, it does not show the same pattern as observed in most species of Neotropical Psittacidae (Seabury et al., 2013; Furo et al., 2015a, 2018). Moreover, it was possible to confirm only one chromosome signature of Psittaciformes in the karyotype of this species, in this case, the fusion between PAK8(GGA8)/PAK9 (GGA9), a feature present in most species of this group, except for two species described recently: *A. aestiva* and *Pyrrhura frontalis* (Furo et al., 2018).

Thus, based on the chromosome painting data, Furo et al. (2018), considered the existence of two groups of Neotropical Psittacidae, according to the presence or absence of specific chromosomal synapomorphies, such as the association between PAK1/PAK4 (GGA1p/GGA4q), PAK6/PAK7 (GGA6/GGA7) and PAK8/PAK9 (GGA8/GGA9). In this case, *M*. *monachus* shows chromosomal characteristics found in both groups.

The role of microchromosomes in the karyotype evolution of birds has proven to be one of the greatest enigmas in the comparative chromosome analyses of birds (O’Connor et al., 2018). In general, the available chromosome painting probes correspond only to the first ten pairs of chicken chromosomes, which can be sorted by flow cytometry due to their differences in size and GC content (Yang et al., 1999). However, the analyses of microchromosomes are essential to draw a more accurate phylogenetic picture, especially in groups with low diploid numbers and atypical karyotypes, such as the Falconiformes and Psittaciformes. The introduction of comparative chromosome mapping of chicken BACs has helped to resolve this difficulty. Despite the constant chromosome complement (2*n* = 70) in most members of the Arini Tribe, it has been observed that several macrochromosome regions were not hybridized by any of the GGA whole-chromosome probes, which could indicate cryptic fusions with microchromosomes (Furo et al., 2015a, 2018; O’Connor et al., 2018). This conclusion is confirmed in *M*. *monachus* where many tandem fusions between microchromosomes and macrochromosomes were observed, reducing the diploid chromosome number of this species to 2*n* = 48.

### Phylogenetic Relationship in Neotropical Psittacidae

Recent analyses of mDNA and nuclear genes have started to resolve the phylogenetic relationships in the Neotropical Psittacids, and comparative chromosome painting has helped also in the understanding of karyotype evolution in this group.

Tavares et al. (2006) in their study with nuclear and mitochondrial markers in 25 of the 30 genera of Neotropical Psittacidae suggested the division of this group into three large clades, which did not correspond to the grouping by tail size (short and long tail) as proposed by Sick (1997). In the proposal of Tavares et al. (2006), *M*. *monachus* is placed in a basal position in the phylogenetic tree, together with Amazonian species and allies of the genera: *Pionus*, *Graydidascalus*, *Pionopsitta*, *Tricalia* and *Botogeris*. These results are consistent with studies by Wright et al. (2008) who used multilocus molecular character sampling (3,941 bp from mitochondrial DNA (mtDNA) genes, cytochrome oxidase I and NADH dehydrogenase 2 and nuclear introns of rhodopsin intron 1, tropomyosin alpha-subunit intron 5, and transforming growth factor ss-2).

**FIGURE 10:**
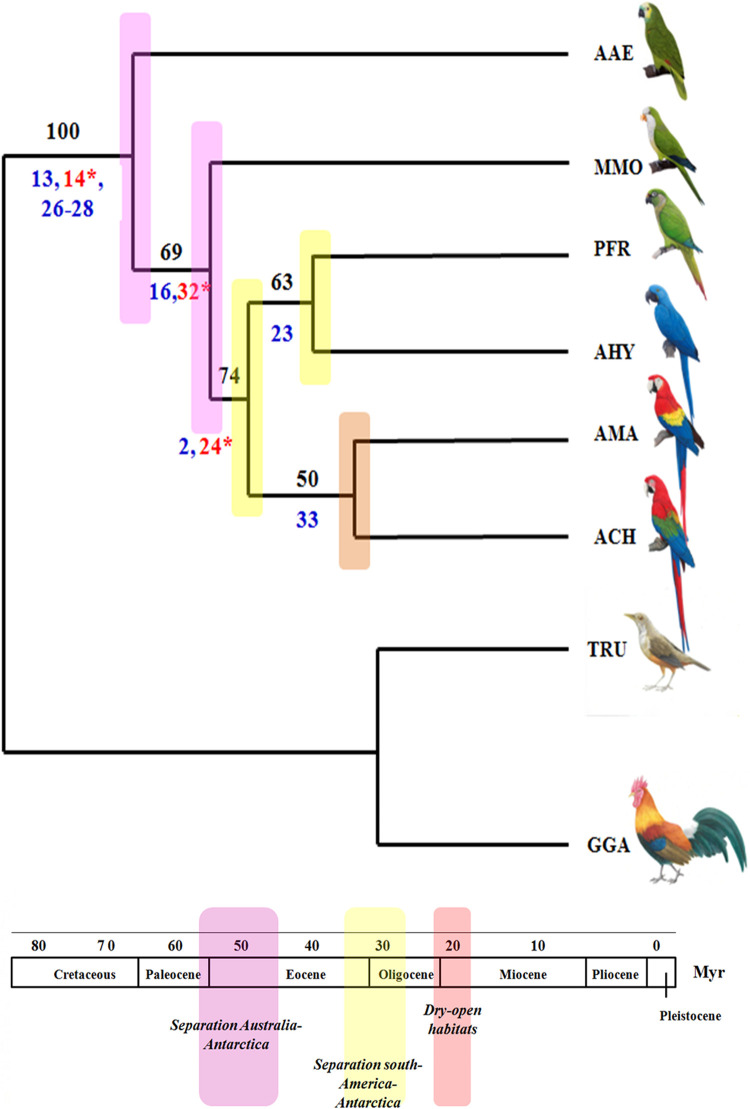
Phylogenetic analysis of Maximum Parsimony made using PAUP based on chromosome rearrangements present in Neotropical Psittacidae, according to results obtained by FISH from *G. gallus* probes (Legend: AMA, *Ara macao*, ACH, *Ara chloropterus*, AHY, *Anodorhynchus hyacinthinus*, PFR, *Pyrrhura frontalis*, AAE, *Amazona aestiva*, GGA, *Gallus gallus*, TRU, *Turdus rufiventris*, MMO, *Myiopsitta monachus*, Myr, millions of years). The red numbers correspond to features that appear in several clades, however, the blue numbers correspond to the characteristics that place the clades together; the bootstrap numbers are in black. The estimation of geological events was based on the literature review by Tavares et al. (2006).

In our analysis of maximum parsimony, it was possible to clarify doubts about the phylogenetic position of *M*. *monachus*. Although this species has a long tail and a karyotype consisting mainly of metacentric and submetacentric chromosomes, as well as the association of PAK8/PAK9, our analysis placed this species in a basal position, corresponding to a second radiation, after *A*. *aestiva*. This result agrees with previous analysis performed with mDNA and nuclear sequences (Tavares et al., 2006; Wright et al., 2008). In this analysis, we also found that the main characters that united the macaws to a common ancestor, is the association of PAK4q/PAK1p (GGA4q/GGA1p), in addition to a fusion between PAK5 (GGA5) with a microchromosome (Characters 2, 24). As proposed by us previously (Furo et al., 2018), the fusion between PAK4q/PAK1p (GGA4q/GGA1p) was already present in the common ancestor of the macaws (Table 5 and Figure 10).

**TABLE 5:** Correspondence between syntenic groups of Psittaciformes species analyzed by FISH and the putative ancestral avian karyotype (PAK) and *Gallus gallus* chromosomes (GGA).

Species	Chromosomes											2n	Distribution	References
GGA	1	2	3	4q	5	6	7	8	9	4p	10	78		Griffin et al., 2007
PAK	1	2	3	4	5	6	7	8	9	10	11	80		Griffin et al., 2007
AHY	1q/4	2	3	1p	5	6q	6q	7p	7q	8q	9q	70	Neotropical	Furo et al., 2015a
ACH	1q/4	2/11	3	1p	5q	6q	6q	7pq	7q	8q/10q	9	70	Neotropical	Furo et al., 2015a
PFR	1q/4	2	3	1p	5q	6q	6q	7	8	10	9	70	Neotropical	Furo et al., 2018
AMA	1q/4/9q	2	3	1p	5q	6q	6q	7pq	7q	8q		70	Neotropical	Seabury et al., 2013
MMO	3q,4p/q,12,14q	2,3p,13q	5,7	1q	6	9q,10q	9q,10q	8p	8q	1p/7q	9p,11	48	Neotropical	Present work
AAE	2/5q	1/12	3	4q	6	7q	7q	11	8	10	9p	70	Neotropical	Furo et al., 2018
PER	1q/4	2	3	4q	5q	6q	6q	7q	7q	micro		70	African	Furo et al., 2018
ARO	3/4q	2/9q	1	7	8q	6q	6q	5q	5q/9q	4p	10	48	African	Nanda et al., 2007
MUN	3/6	1	2	7	4q	4p/8p	4p	5pq	5q	5p	9q	62	Australia	Nanda et al., 2007
NHO	3/6	1	2	4	7q	5	5	4p	4p/10	11	9	72	Australia	Nanda et al., 2007

*GGA (Gallus gallus), PAK (Putative ancestral Karyotype), AHY (Anodorhynchus hyacinthinus), ACH (Ara chloropterus), PRF (Pyrrhura frontalis), AMA (Ara macao), MMO (Myiopsitta monachus), AAE (Amazona aestiva), PER (Psittacus erithacus), ARO (Agapornis roseicollis), MUN (Melopsittacus undulatus), NHO (Nymphicus hollandicus).*

In an attempt to improve the phylogenetic tree, the conflicting character corresponding to the fusions between PAK4 (GGA4p) with microchomosomes – shared by ACH, AMA, and AAE – was removed from the binary matrix. This feature could support the relationship between AMA and ACH, however, only analysis with chicken BACs from microchomosomes could clarify whether these fusions involve the same microchromosomes. The use of chicken BACs in different species would help to improve support of the different branches in the phylogenetic tree.

Despite the high number of chromosomal rearrangements, most of them corresponded to autapomorphisms (Supplementary Table S1, Characters – 8–12, 17–20, 30,31). Because of the low number of informative chromosomal characters, chromosome painting data did not throw any light on the time of divergence of the species analyzed. However, consideration of our phylogenetic proposal and comparisons with previously published data, led us to assume that the common ancestor of the clade of *M*. *monachus* and phylogenetically related genera (for example, *Amazona*) could have been originated in the Eocene around 48 Mya. This was a period of major changes in South America, including drastic changes in temperature due to the separation of Australia-Antarctica caused by continental drift, which changed the fauna and flora scenario around 32 Mya. The ancestor of *M*. *monachus* would have appeared during the separation of South America and Antarctica (Tavares et al., 2006).

### Significance of Whole Chromosome Painting With MMO Probes

Interchromosomal rearrangements have played an important role during the karyotype evolution of the Psittaciformes (Nanda et al., 2007; Seabury et al., 2013; Furo et al., 2015a, 2018). As mentioned before, *M*. *monachus* showed several fissions involving pairs corresponding to PAK1 (GGA1), PAK2 (GGA2), PAK3 (GGA3), PAK6 (GGA6), PAK7 (GGA7), e PAK10 (GGA4p). In this context, this species becomes interesting from the cytogenetic point of view. Hence, with the analysis of LAL probes in the metaphases of MMO, it was possible to check many breakpoints that were not recurrent. Furthermore, the utilization of these probes onto other species of Psittaciformes will be very useful for genomic comparisons, because the signals of hybridization are more evident due to phylogenetic proximity.

MMO probes were used in metaphases of *A*.*macao* and produced good signals which showed a fusion between MMO10 (microchromosome) with PAK5 (GGA5). Generally, the GGA5 probe does not hybridize to the entire length of particular chromosome pair in macaws and parrots (Furo et al., 2015a, 2018) and a gap was always observed, leading authors to suppose it corresponded to a fusion with a microchromosome. It would be interesting to use the same probes in other species of Neotropical Psittacidae to check for the same association.

## Conclusion

The description of the *M*. *monachus* karyotype using different sets of chromosome probes showed that, although most species of Neotropical Psittacidae have a constant diploid chromosome number of 2*n* = 70 and a similar karyotype in terms of uni- and biarmed elements, this species shows high rate of karyotype repatterning, with 2*n* being reduced to 48 chromosomes. *M*. *monachus* is the first to have its karyotype described in detail, with probes corresponding to macro and microchromosomes, revealing many inversions and fissions, which together with no-reciprocal translocations and tandem fusions represent the main karyotype rearrangements in Psittaciformes. Additionally, although this species has a long tail, our phylogenetic analysis placed it in a basal position together with *A*. *aestiva* (short tail), mainly due the low number of chromosome synapomorphies, as most of the numerous rearrangements corresponded to autapomorphies. Thus, our results corroborate previous studies performed by mDNA and nuclear sequences. Moreover, we have shown that the MMO probes are useful tools in the analysis of evolutionary chromosome rearrangements, because they reveal novel breakpoints previously undescribed in the literature.

## Data Availability Statement

All datasets generated for this study are included in the article/Supplementary Material.

## Ethics Statement

The animal study was reviewed and approved by CEUA-Universidade Federal do Pará, no. 170/2013.

## Author Contributions

IF and EO: conceptualization, validation, and writing (original draft). IF, RK, AG, and EO: data curation and formal analysis. IF, RK, AG, RO’C, AG, and RG: investigation. IF, PO’B, and JP: methodology. EO: project administration. MF-S, RG, AG, and EO: funding acquisition. PO’B and MF-S: writing (review and editing). All authors corrected, revised, and discussed the data.

## Conflict of Interest

The authors declare that the research was conducted in the absence of any commercial or financial relationships that could be construed as a potential conflict of interest.
