# Supplementary material for: Chromosomal Evolution in the Phylogenetic Context: A Remarkable Karyotype Reorganization in Neotropical Parrot Myiopsitta monachus (Psittacidae)
Source: Front Genet. 2020 Jul 10;11:721. doi: 10.3389/fgene.2020.00721 (PMC7366516; doi:10.3389/fgene.2020.00721)
Supplement: Supplementary file 1 [file Table_1.DOCX]

Table S1- Data matrix used in the PAUP phylogenetic program.. The abbreviations correspond to chromosome rearrangements detected with *Gallus gallus* probes: FUS_Fusion; FIS_Fission; PA_Paracentric inversion; PE_Pericentric Inversion; dist_ distal chromosome segment; Prox_ proximal centromere region; p_short arm; q_ long arm. Scientific name abbreviation: GGA_ *Gallus gallus*; TRU_ *Turdus rufiventris*; AHY_ *Anodorhynchus hyacinthinus*; ACH_ *Ara chloropterus*; AMA_ *Ara macao*; PFR_ *Pyrrhura frontalis*; AAE_ *Amazona aestiva*; MMO_ *Myiopsitta monachus*.

| No. | CHARACTERES GGA | GGA  2n=70 | TRU  2n=78 | AHY  2n=70 | ACH  2n=70 | AMA  2n=68 | PFR  2n=70 | AAE  2n=70 | MMO  2n=48 |
| --- | --- | --- | --- | --- | --- | --- | --- | --- | --- |
| 1 | FUS GGA1 | 1 | 0 | 0 | 0 | 0 | 0 | 0 | 0 |
| 2 | FUS GGA1p/4q | 0 | 0 | 1 | 1 | 1 | 1 | 0 | 0 |
| 3 | FIS GGA 1q | 0 | 0 | 0 | 0 | 1 | 0 | 0 | 0 |
| 4 | FIS GGA 1 | 0 | 0 | 0 | 0 | 0 | 0 | 1 | 0 |
| 5 | FUS GGA 1q-dist/micro | 0 | 0 | 0 | 0 | 1 | 0 | 0 | 0 |
| 6 | FUS GGA1q-prox./micro | 0 | 0 | 0 | 0 | 0 | 0 | 1 | 0 |
| 7 | FUS GGA1p-dist/micro | 0 | 0 | 0 | 0 | 0 | 0 | 0 | 1 |
| 8 | FUS GGA 1q/micro | 0 | 0 | 0 | 0 | 0 | 0 | 0 | 1 |
| 9 | FUS GGA 1q/GGA2 | 0 | 0 | 0 | 0 | 0 | 0 | 0 | 1 |
| 10 | FUS GGA1q/micro | 0 | 0 | 0 | 0 | 0 | 0 | 0 | 1 |
| 11 | FIS GGA1q as in MMO12 | 0 | 0 | 0 | 0 | 0 | 0 | 0 | 1 |
| 12 | FIS GGA1q as in MMO14 | 0 | 0 | 0 | 0 | 0 | 0 | 0 | 1 |
| 13 | GGA2 as in GGA | 1 | 1 | 0 | 0 | 0 | 0 | 0 | 0 |
| 14 | FIS GGA2p | 0 | 0 | 0 | 1 | 0 | 0 | 1 | 1 |
| 15 | FUS GGA2p/micro | 0 | 0 | 0 | 0 | 0 | 0 | 0 | 1 |
| 16 | PE GGA3 | 0 | 0 | 1 | 1 | 1 | 1 | 0 | 1 |
| 17 | FIS GGA3 as in MMO | 0 | 0 | 0 | 0 | 0 | 0 | 0 | 1 |
| 18 | FUS GGA3/micro as in MMO5 | 0 | 0 | 0 | 0 | 0 | 0 | 0 | 1 |
| 19 | FUS GGA3/micro as in MMO7 | 0 | 0 | 0 | 0 | 0 | 0 | 0 | 1 |
| 20 | FUS GGA3/GGA 4 as in MMO7 | 0 | 0 | 0 | 0 | 0 | 0 | 0 | 1 |
| 21 | FUS GGA4p/q | 1 | 0 | 0 | 0 | 0 | 0 | 0 | 0 |
| 22 | FIS GGA4p | 0 | 0 | 0 | 1 | 0 | 0 | 0 | 0 |
| 23 | PE GGA4p | 0 | 0 | 1 | 0 | 0 | 1 | 0 | 0 |
| 24 | FUS GGA 5/micro | 0 | 0 | 1 | 1 | 1 | 1 | 0 | 0 |
| 25 | FIS GGA 6/7 | 1 | 1 | 0 | 0 | 0 | 0 | 0 | 0 |
| 26 | FUS GGA 6/7 | 0 | 0 | 1 | 1 | 1 | 1 | 1 | 1 |
| 27 | PA GGA 7:6:7:6 | 0 | 0 | 1 | 1 | 1 | 1 | 1 | 1 |
| 28 | FUS GGA 7-6-7-6/micro | 0 | 0 | 1 | 1 | 1 | 1 | 1 | 1 |
| 29 | FUS GGA 7-6-7-6/micro | 0 | 0 | 0 | 0 | 0 | 0 | 1 | 0 |
| 30 | FUS 8-3/micro | 0 | 0 | 0 | 0 | 0 | 0 | 0 | 1 |
| 31 | FUS 10/ 8-3 | 0 | 0 | 0 | 0 | 0 | 0 | 0 | 1 |
| 32 | FUS GGA 8/9 | 0 | 0 | 1 | 1 | 1 | 0 | 0 | 1 |
| 33 | PE GGA 8-9-8 | 0 | 0 | 0 | 1 | 1 | 0 | 0 | 0 |
| 34 | FUS GGA 9/MICRO | 0 | 0 | 0 | 0 | 0 | 0 | 1 | 0 |
